# The Value of Cutting Seton for High Transsphincteric Anal Fistula in the Era of Its Misery

**DOI:** 10.21315/mjms2022.29.1.6

**Published:** 2022-02-23

**Authors:** Abdel Latif Khalifa Elnaim Ali, Michael Pak-Kai Wong, Ismail Sagap

**Affiliations:** 1 Department of Surgery, Kassala Police Hospital, Kassala, Sudan; 2 Department of Surgery, School of Medical Sciences, Universiti Sains Malaysia, Kelantan, Malaysia; 3 Department of Surgery, Universiti Kebangsaan Malaysia Medical Centre, Kuala Lumpur, Malaysia

**Keywords:** transsphincteric fistula, cutting seton, incontinence, fistula recurrence, fistula outcome

## Abstract

**Objectives:**

We studied the outcome of cutting seton in the treatment of a high transsphincteric anal fistula in Sudan.

**Methods:**

This was a prospective study of high transsphincteric anal fistulas at Kassala Police Hospital, Sudan, over the course of 24 months (2016–2017). The main outcomes measured were recurrence, incontinence and primary healing rates.

**Results:**

The cases of 72 patients treated with cutting seton for high transsphincteric fistula were analysed, with 50 (70%) of the patients being male and 22 (30%) being female. Forty-eight (66.7%) patients required two sessions of seton tightening with a duration of seton treatment of 30 days and 24 (33.3%) patients required three sessions with a duration of seton treatment of 45 days. Only one patient (1.4%) had flatus incontinence. Three (4.2%) patients had minimal bleeding from the seton site and two (2.8%) patients experienced fistula recurrence. Twenty-six (36%) patients achieved complete healing within 30 days, while 36 (54.3%) patients healed within 60 days. The remaining seven (9.7%) patients healed after 60 days. Chronic pain was reported by two (2.8%) patients after complete healing.

**Conclusion:**

In Sudan, cutting seton remains relevant, as it produces minimal incontinence with a low recurrence rate in high transsphincteric fistula treatment.

## Introduction

A fistula is an abnormal epithelised communication between two epithelial surfaces. An anal fistula or fistula-in-ano connects the anorectum to the perineal skin. Fistula-in-ano is one of the most common surgical problems, with an incidence rate of up to 2.8 per 10,000 (1). Such conditions usually affect men more than women and those affected are most often in their fifth decade of life (2–5). The usual presentation of this condition involves intermittent pain, itching and the discharge of pus, blood or faeces (2).

A high transsphincteric anal fistula has been defined as a fistula tract passing through the upper or middle third of the external anal sphincter or mid-anal canal (6, 7). Cutting seton is a suture knot that is placed in the fistulous track around the anal sphincter that is tied with tension and tightened intervally until the track is completely superficialised or cut through. Cutting seton has been associated with high faecal incontinence with a reported rate of 25% (2). This is considerably worrying; however, this technique is easy to learn and can be performed by general surgeons.

In Kassala, Sudan, cutting seton has been used by most general surgeons for the treatment of high transsphincteric anal fistulas despite concerns about such an intervention leading to incontinence. In our study, we evaluated the outcomes of cutting seton for the treatment of high transsphincteric anal fistulas over the course of 12 months at Kassala Police Hospital.

## Methods

**Figure 1:**
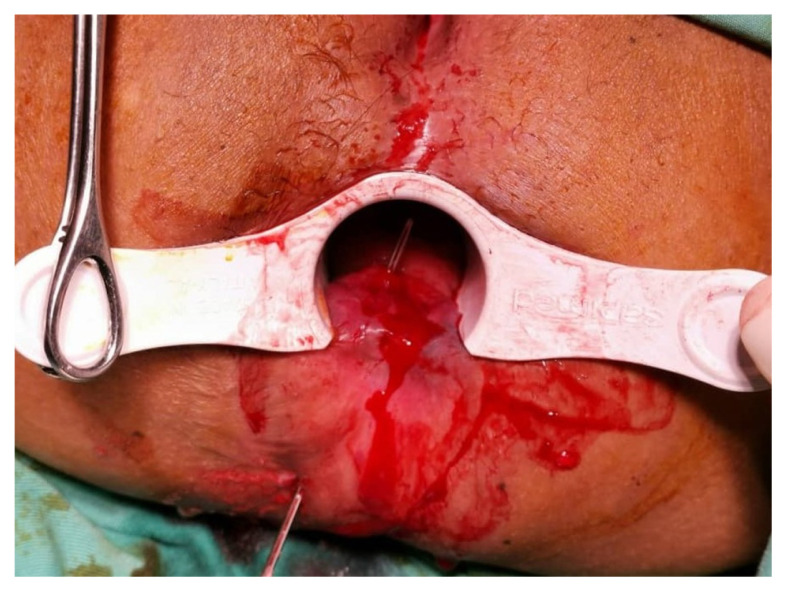
High transphincteric anal fistula as the internal opening located at the mid-anal canal and the tract traverse middle (beyond 30%) of the external anal sphincter

This was a prospective study conducted at Kassala Police Hospital, Sudan, from 2016 to 2017 after approval from our institution review board. All the patients were above 18 years old of age and were diagnosed with high transsphincteric anal fistula during the recruitment period. The high transsphincteric fistula was described as a fistula that passes at the mid-anal canal and traverses across the middle or upper external anal sphincter. We anatomically divided the external anal sphincter into three parts in relation to the anal canal. The transsphincteric fistula was gauged via digital rectal examination and deemed a high transsphincteric fistula when the tract traversed beyond 30% of the external anal sphincter (Figure 1). Patients who had pre-existing conditions such as Crohn’s disease, tuberculosis, malignancy, human immunocompromised virus (HIV) infections, recurrent fistula or complex fistula were excluded, as were those who refused to provide informed consent. Before the operations, their status regarding incontinence was determined via a simple question about the presence of faecal or flatus incontinence. The same question was later repeated after surgery at the outpatient follow-up. All patients were followed up post-operatively at the surgical outpatient clinic for at least 12 months. Outcome measures were specifically asked about or reported voluntarily by the patients during their follow-ups.

**Figure 2:**
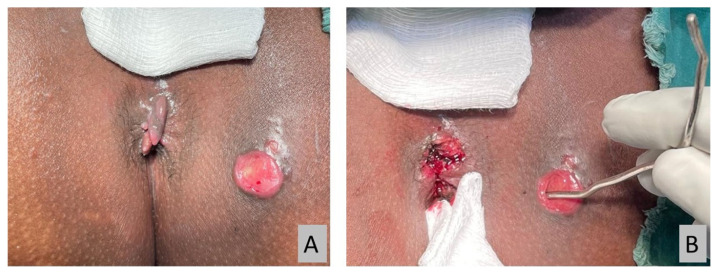
The fistula probe is used to locate the direction of the tract and facilitate insertion of the cutting seton with Silk 2 sutures

**Figure 3:**
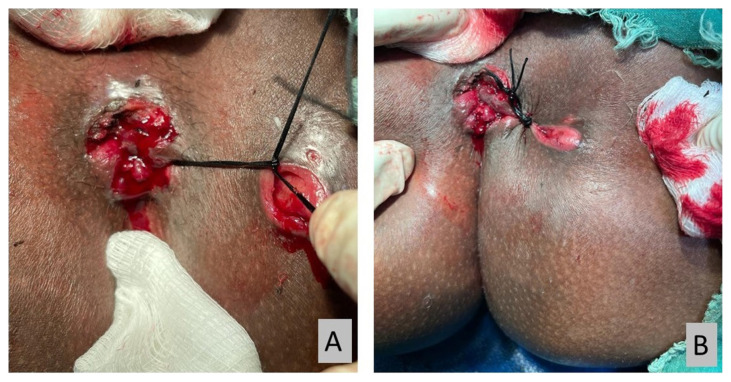
The Silk 2 sutures have been threaded through and tightened with double knots to ensure secure knots

**Figure 4:**
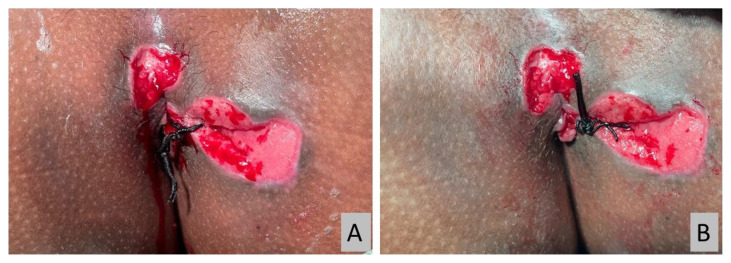
(A) The loosened cutting seton after two weeks, (B) the re-tightened cutting seton using a new suture

All operations were performed under spinal anaesthesia by a single surgeon. Examinations were conducted with patients under anaesthesia to confirm the pre-operative diagnosis (Figure 2). Silk suture size 2 (Silk 2 suture) was used as a cutting seton (Figure 3). Silk suture was preferred as it is a non-absorbable multifilament type; therefore, the knots hold better and loosen negligibly over time. It also creates a more intense tissue reaction for fibrosis while cutting through the tract. All patients had an adjustment session every 2 weeks until the complete cut-through was achieved in a minor procedure room under sedation (Figure 4).

The numerical data were reported as medians and interquartile ranges, and the categorical data were reported as frequencies and percentages.

## Results

Seventy-two patients diagnosed with high transsphincteric anal fistulas in the Kassala Police Hospital underwent treatment between 2015 and 2017. There were 50 male (70%) and 22 female (30%) patients. Forty-eight patients (66.7%) needed two sessions of seton tightening over a 30-day duration, while 24 patients (33.3%) required three sessions over 45 days. During the post-operative follow-up, there was no reported faecal incontinence. However, flatus incontinence was reported by one patient. This condition developed in a patient who required three sessions of seton tightening. Fistula recurrence was observed in two (2.8%) patients after 6 months of follow-up. Primary healing was achieved in 62 (90.3%) patients within 60 days. The remaining seven (9.7%) patients required more than 60 days to heal.

Regarding other complications, minimal bleeding developed in three patients (4.2%), which did not require surgical intervention, while pruritus ani with perianal discharge was observed in four patients (6.9%). In addition, two patients (2.8%) experienced chronic anal pain. The pain in these cases was described as minimal and tolerable after primary healing was achieved (Table 1).

## Discussion

Anal fistulas represent an essential aspect of colorectal practice, emerging as a distressing condition for patients and sometimes imposing a challenge upon surgeons. They have been classified into four significant types according to their relation to the anal sphincter complex, which include intersphincteric, transsphincteric, suprasphincteric and extrasphincteric (3, 8). This classification is a means of helping to improve the management plan. Treatment strategies should incorporate a closure of the internal opening and an excision of the infected fistula tract with preservation of the sphincter complex (3, 5, 8–10).

Radical excisional treatments such as fistulectomies (removal of the fistula tract) or fistulotomies (laying open of the fistula tract) in high anal fistulas have had an overall post-operative incontinence rate of 35%. At the same time, non-radical modalities, such as the use of fibrin glue and advancement flap repair, have had recurrence rates of 40% and 46%, respectively (11). To date, fistulotomies and fistulectomies produce the best healing rates and have become the standard of treatment. These success rates reflect the fact that the high transsphincteric fistula type is indeed vulnerable to the highest risk of sphincter damage during treatment (2, 3, 9, 10).

Alternative methods that have been introduced to preserve the anal sphincter include the use of setons, fibrin glue, collagen plugs and the rectal mucosa advancement flap (2, 3). Furthermore, as of late, ligation of the intersphincteric fistula tract (LIFT), the Bio-LIFT procedure, fistula tract laser closure (FiLaC), video-assisted anal fistula (VAAFT) treatment and adipose-derived stem cell treatment are sphincter preserving options that have had reasonable healing and significantly low incontinence rates (12). However, these methods are rather expensive options and require highly skilled colorectal surgeons, and such individuals are not yet readily available in a developing country like Sudan.

Setons are used as a treatment option for high transsphincteric fistulae and in patients with a high probability of developing incontinence (4, 8, 9, 13). A seton is defined as any string-like material or physical agent that is inserted into the fistula tract, and it is a cutting seton when it is tied along the fistula tract to gradually cut through the sphincter complex, leading to an inflammatory reaction, which eventually gets fibrosed. In contrast, a draining seton is a loosely tied seton along the fistula tract that allows for the maturation of the track and drainage of intersphincteric collections. Cutting seton application aims for the preservation of the sphincter through a gradual, staged fistulotomy (3, 8–10).

Commonly used materials for setons are non-absorbable sutures like Prolene, Penrose drains, rubber bands, vessel loops and silastic catheters. A medicated Ayurveda thread called Kshar Sutra was the earliest known seton described by Sushruta, which consisted of linen thread soaked in kshara, an alkaline chemical derived from plant extracts with a slight tissue cutting property (3, 8, 9, 13). The core principle of cutting seton involves striking a balance between the degree of the sphincter’s pressurised cut and primary wound healing with the preservation of anal sphincter function (14). In the past, cutting seton had fallen out of favour because of its implementation, resulting in a high incontinence rate of about 12% to 18% (15). Therefore, the trend has been turning towards the use of other modalities to minimise incontinence in the treatment of anal fistulas.

In this study, treatment with cutting seton exhibited a low incontinence complication rate of 1.4%. This could be due to the patient’s perceived higher expectation for healing and the lower expectation of incontinence during this treatment. This may explain the lower number of reported cases of incontinence among our cohort. Further, the cases of incontinence presented were minor flatus incontinence that did not require any surgical re-intervention. It was also found that chronic pain was experienced by two patients. The pain was described as tolerable and minimal, and these cases were presented after complete healing of the fistula within 45 days.

Anal fistula treatment must navigate through the complex terrain of both the patient’s expectations and the surgeon’s experience. The surgeon should consider the trade-off between the extent of sphincter division with a degree of functional loss and the primary healing rate (8).

The cutting seton used in our patients was Silk 2 sutures, and it was tightened every two weeks to steadily and gradually cut along the tract with minimal complications. This technique has been used in our centre for more than 10 years. We have also observed a recurrence rate of 2.8% and there was no patient with faecal incontinence after 12 months of follow-up. The other reported incidence rates of incontinence after cutting seton ranged from 0% to 62% (Table 2) (1, 8, 16–19). Our data have shown reasonably good results for the preservation of sphincter function.

## Limitations and Recommendations

Our study was limited by a smaller sample size. Furthermore, we did not routinely use incontinence score tools, which may be helpful to gauge the severity of cases of incontinence. We did not explore these issues further due to the low incidence rate of incontinence. It is also important to note that in our cohort, we did not routinely use imaging for new cases of anal fistula due to financial constraints. Perhaps in future studies, standardised incontinence scores and imaging, such as endoanal ultrasound, could be used to study this more objectively. To increase the sample size, multi-centre recruitment and a longer recruitment study period would also be recommended.

## Conclusion

The use of the cutting seton technique by general surgeons in Sudan has been shown to be a good option for treating high transsphincteric anal fistulas with generally positive results and low incontinence rates.

## Figures and Tables

**Table 1:** Demographic data, complications and recurrence rates

Demographic data	
Total patients	72
Age (median; range) (years old)	46 (20–66)
Gender (*n*/%)	
Female	50 (69.4)
Male	22 (30.6)

**Complications (** ***n*** **/%)**	

Flatus incontinence	1 (1.4)
Bleeding	3 (4.2)
Pruritus ani	5 (6.9)
Chronic anal pain	2 (2.8)

**Time to wound healing**	

1 week	26 (36.1%)
2 weeks	39 (54.2%)
More than 2 weeks	7 (9.7%)

Recurrence	2 (2.8%)

**Table 2:** Summary of studies on recurrence and incontinence rates

Author (Year)	No. of patients	Recurrence rate (%)	Incontinence rate (%)
García-Aguillar et al. (1998) (16)	12	8.3	66.7
Chuang-Wei et al. (2008) (17)	112	0.9	24.1
Kamrava and Collins (2011) (18)	47	9.0	2.0
Memon et al. (2011) (8)	79	5.1	0
Raslan et al. (2016) (19)	51	9.8	21.3
Al-Marzooq et al. (2017) (1)	55	2.9	13.2
Abdel Latif et al. (current study)	72	2.8	1.3
